# The Many Faces of Matrix Metalloproteinase-7 in Kidney Diseases

**DOI:** 10.3390/biom10060960

**Published:** 2020-06-25

**Authors:** Zhao Liu, Roderick J. Tan, Youhua Liu

**Affiliations:** 1 State Key Laboratory of Organ Failure Research, National Clinical Research Center of Kidney Disease, Nanfang Hospital, Southern Medical University, Guangzhou 510515, China; lz0103@i.smu.edu.cn; 2 Renal-Electrolyte Division, Department of Medicine, University of Pittsburgh School of Medicine, Pittsburgh, PA 15261, USA; tanrj@upmc.edu; 3 Department of Pathology, University of Pittsburgh School of Medicine, Pittsburgh, PA 15261, USA

**Keywords:** matrix metalloproteinase-7, fibrosis, proteinuria, acute kidney injury, chronic kidney disease, apoptosis

## Abstract

Matrix metalloproteinase-7 (MMP-7) is a secreted zinc-dependent endopeptidase that is implicated in regulating kidney homeostasis and diseases. MMP-7 is produced as an inactive zymogen, and proteolytic cleavage is required for its activation. MMP-7 is barely expressed in normal adult kidney but upregulated in acute kidney injury (AKI) and chronic kidney disease (CKD). The expression of MMP-7 is transcriptionally regulated by Wnt/β-catenin and other cues. As a secreted protein, MMP-7 is present and increased in the urine of patients, and its levels serve as a noninvasive biomarker for predicting AKI prognosis and monitoring CKD progression. Apart from degrading components of the extracellular matrix, MMP-7 also cleaves a wide range of substrates, such as E-cadherin, Fas ligand, and nephrin. As such, it plays an essential role in regulating many cellular processes, such as cell proliferation, apoptosis, epithelial-mesenchymal transition, and podocyte injury. The function of MMP-7 in kidney diseases is complex and context-dependent. It protects against AKI by priming tubular cells for survival and regeneration but promotes kidney fibrosis and CKD progression. MMP-7 also impairs podocyte integrity and induces proteinuria. In this review, we summarized recent advances in our understanding of the regulation, role, and mechanisms of MMP-7 in the pathogenesis of kidney diseases. We also discussed the potential of MMP-7 as a biomarker and therapeutic target in a clinical setting.

## 1. Introduction

Matrix metalloproteinase-7 (MMP-7), also known as matrilysin-1, is one of the smallest secreted proteases of the MMP family, which consists of more than 20 structurally-related zinc-dependent endopeptidases with broad substrate specificity [1,2]. Collectively, MMPs degrade virtually all kinds of extracellular matrix (ECM) proteins and contribute to their turnover and remodeling. Furthermore, MMPs can also cleave many non-ECM substrates, making them a critical player in a wide variety of physiologic and pathologic processes, such as cell proliferation and apoptosis, endothelial cell function, inflammation, and tumor metastasis and invasion [3,4].

Extensive studies have shown that many MMPs are implicated in regulating kidney development and the pathogenesis of kidney diseases. Dysregulation of MMPs has been described in a wide variety of kidney disorders, including acute kidney injury (AKI), chronic kidney disease (CKD), diabetic kidney disease (DKD), glomerulonephritis, and inherited kidney diseases. Over the last two decades, due to the availability of MMP knockout models and specific pharmacological inhibitors, the role of MMPs, particularly MMP-2 and MMP-9, in regulating the development and progression of kidney diseases have been extensively investigated. The detailed discussion of these MMPs in kidney pathology as well as in non-renal diseases is beyond the scope of the present paper, and the interested readers are referred to several comprehensive reviews on this topic [1,2,5]. Instead, the focus of this review is on MMP-7 in kidney diseases. 

MMP-7 was first discovered in the rat uterus [6]. Structurally, MMP-7 comprises a pro-peptide domain and a catalytic domain. While it was formerly believed to cleave only ECM proteins, the evidence is emerging that MMP-7 also degrades a variety of non-ECM substrates, such as nephrin, E-cadherin, Fas ligand (FasL), pro-MMP-2, and pro-MMP-9 [7,8,9,10]. As such, MMP-7 is able to regulate a diverse array of biological processes, such as podocyte dysfunction, epithelial to mesenchymal transition (EMT), cell proliferation, and apoptosis [11,12]. It should be stressed that many actions of MMP-7 in the kidney are highly specific and unique. For instance, only MMP-7, but neither MMP-2 nor MMP-9, can cleave podocyte slit diaphragm protein nephrin and cause proteinuria [7]. In this context, it is conceivable that MMP-7 may be more important than other MMPs to the pathogenesis of kidney diseases.

In the past several years, significant progress has been made in our understanding of the biology of MMP-7 in kidney diseases. Numerous novel substrates of MMP-7 have been identified, enabling us to better comprehend the exact role of MMP-7 in the pathogenesis of kidney disorders. Mounting evidence suggests that urinary MMP-7 (uMMP-7) can be utilized as a noninvasive biomarker for predicting AKI prognosis and monitoring CKD progression in patients [13,14,15]. In this review, we discussed the expression, regulation, novel substrates, and mechanisms of MMP-7 in various kidney diseases.

## 2. MMP-7 Structure, Activation, and Regulation

**Figure 1:**
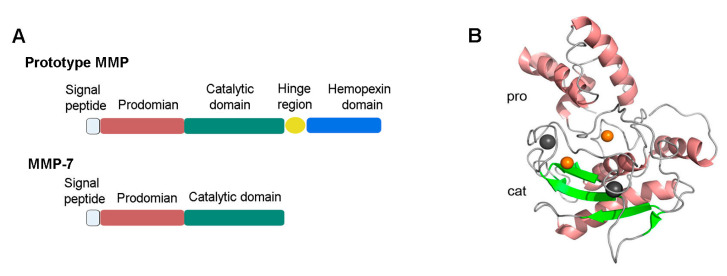
Structure of proMMP-7. (**A**) Full-length proMMP7 only consists of two domains: a pro-peptide domain (pro) and a catalytic domain (cat), which separates it from the prototype of MMPs. (**B**) The pro-peptide domain consists of three α-chains and connecting loops. The catalytic domain contains two zinc ions, two copper ions, and a ball-like structure consisting of three α-helices, five β-sheets, and multiple loops [16]. The image was prepared from Protein Data bank entries 2MZE (proMMP-7) using the PyMol (http://www.pymol.org). MMP, matrix metalloproteinase.

The human *MMP-7* gene is located on chromosome 11 q22.3. The cDNA of *MMP-7* encodes a protein containing 267 amino acids. Structurally, MMP-7 only consists of a pro-peptide domain and a catalytic domain (Figure 1A), which separates it from most other MMPs that contain an additional hinge region and a hemopexin-like domain [1,2,16]. The crystal structure of MMP-7 containing the two domains aforementioned is shown in Figure 1B. 

MMP-7 protein is produced and secreted as an inactive zymogen, which is maintained by a conserved cysteine residue that interacts with the zinc in the active site, rendering the protease inactive [16]. Disruption of this so-called cysteine switch is required for activation and can occur via proteolytic cleavage by many proteases, including trypsin, plasmin, or even other MMPs [17]. To generate a functional MMP-7 from the zymogen, the pro-peptide domain is proteolytically degraded in a stepwise manner [18]. The latent form of MMP-7 is a 28 kDa protein. After removing an approximately 9 kDa sequence from the pro-peptide domain, the resultant 19 kDa peptide represents the active and functional endopeptidase. MMP-7 is also bound by two calcium ions, which plays an important role in stabilizing the secondary structure of the protein. 

The activity of MMP-7 is regulated by a family of naturally occurring endogenous inhibitors known as tissue inhibitors of metalloproteinases (TIMPs). There are four known TIMPs; however, it remains elusive which TIMP has the greatest specificity for MMP-7. There are also potent inhibitors of MMP-7, such as MMP inhibitor II, that can reversibly block MMP-7 activity. However, the selectivity of these inhibitors for MMP-7 is uncertain, as they often inhibit, to a lesser degree, other MMPs as well. One of the challenges in the field is to develop potent and selective inhibitors that are specific for a given MMP. 

The expression of MMP-7 is transcriptionally regulated by different cues, particularly the Wnt/β-catenin and transforming growth factor-β (TGF-β). The promoter of the human *MMP-7* gene contains a TATA box, an activator protein 1 (AP-1) site, and T cell factor (TCF)-binding elements. The AP-1 binding site is essential for mediating MMP-7 expression in response to growth factors, oncogenes, and phorbol ester, while the TCF-binding elements are responsible for mediating MMP-7 induction by Wnt/β-catenin. As TGF-β is known to activate β-catenin signaling [19], it remains elusive whether TGF-β controls MMP-7 expression directly or via β-catenin indirectly. 

## 3. MMP-7 Expression in the Kidney

MMP-7 is commonly expressed in epithelial cells, including the liver, the ductal epithelium of exocrine glands in the skin, salivary glands, and pancreas, and the glandular epithelium of the intestine and reproductive organ and breast. Under normal physiologic conditions, adult kidney exhibits little MMP-7 expression [12,18,20]. Consistent with this notion, mice with global knockout of *MMP-7* are phenotypically normal, without any renal abnormality [12]. These data suggest that MMP-7 is dispensable for kidney structure and function in basal physiologic conditions. 

The expression of MMP-7 is, however, induced in a wide variety of kidney diseases, including AKI, CKD, glomerular disease, inherited kidney disease, and renal cell carcinoma [11,21,22,23,24], suggesting that MMP-7 induction is a common feature of the kidney after various injuries. The expression and localization of MMP-7 protein in various kidney disorders are summarized in Table 1.

### 3.1. Animal Models

MMP-7 is markedly induced after AKI. In experimental animal models of AKI induced by ischemia-reperfusion injury (IRI), cisplatin, or folic acid, both mRNA and protein levels of MMP-7 are upregulated [11]. Following IRI, MMP-7 protein is mainly expressed and localized in the renal tubular epithelium, particularly in the S3 segment of proximal tubules, the epicenter of kidney injury in this model [11]. These results indicate a spatial correlation of MMP-7 induction with tubular injury and repair and regeneration. At this stage, the exact cues for triggering MMP-7 induction in AKI in vivo remain ambiguous, but it is most likely related to activation of Wnt/β-catenin signaling. In vitro studies show that human kidney proximal tubular cells (HKC-8) increase MMP-7 expression upon stimulation of Wnt/β-catenin, whereas inhibition of Wnt/β-catenin by small molecule inhibitor ICG-001 negates MMP-7 induction [21]. 

Induction of MMP-7 is a common finding in a wide variety of CKD characterized by renal fibrosis. MMP-7 expression is increased in renal tubular epithelia in the mouse model of unilateral ureteral obstruction (UUO) [21]. Similar results are obtained in adriamycin nephropathy, a model of focal and segmental glomerulosclerosis (FSGS). Immunohistochemical staining reveals that MMP-7 expression and Wnt/β-catenin activation are closely correlated in both UUO and adriamycin nephropathy [21]. This result is compatible with the findings in tumors that MMP-7 transcript overlaps with the accumulation of β-catenin protein [25,26]. Therefore, it is conceivable that MMP-7 expression in CKD is causatively linked to the activation of Wnt/β-catenin signaling. 

**Table 1:** Expression of matrix metalloproteinase-7 (MMP-7) in kidney diseases.

Disease	Location	Expression	Ref.
**Animal models**			
Ischemia–reperfusion -induced AKI ^1^	Renal tubular epithelia	Increase	[11]
Folic acid-induced AKI	Renal tubular epithelia	Increase	[11,23]
Cisplatin-induced AKI	Renal tubular epithelia	Increase	[11]
UUO ^2^	Renal tubular epithelia, interstitial cells	Increase	[12,21]
**Human kidney diseases**			
FSGS ^3^	Renal tubular epithelia, interstitial cells, podocytes	Increase	[12,21]
Lupus nephritis	Renal tubular epithelia	Increase	[27]
Membranous nephritis	Renal tubular epithelia	Increase	[12]
Autosomal dominant polycystic kidney disease	Epithelial cells lining cysts, atrophic tubules	Increase	[23]
Diabetic nephropathy	Renal tubular epithelia, interstitial cells	Increase	[12,21]
Hydronephrosis	Cells lining dilated and atrophic tubules	Increase	[23]
Thrombotic microangiopathy	Renal tubular epithelia	Increase	[12]
IgA nephropathy	Renal tubular epithelia, infiltrated inflammatory cells	Increase	[12,21]
Acute renal allograft rejection	Renal tubular epithelia	No change	[28]
Chronic allograft nephropathy	Renal tubular epithelia	Increase	[29]
Amyloid light-chain amyloidosis	Glomerulus, tubular interstitium, vasculatures	Increase	[30]
Light chain deposition disease	Glomerulus, tubular interstitium, vasculatures	No change	[30]
Renal cell carcinoma	Cancer cells and endothelial cells	Increase	[22]

^1^ Acute kidney injury. ^2^ Unilateral ureteral obstruction. ^3^ Focal segmental glomerulosclerosis.

### 3.2. Human Kidney Biopsies

Consistent with animal studies, the induction of MMP-7 expression is also evident in the kidney from patients with various renal disorders. For instance, in the kidney biopsies of patients with an autosomal dominant polycystic kidney disease, MMP-7 staining is observed in tubular epithelial lining cysts and atrophic tubules [23]. In the specimens of patients with hydronephrosis, MMP-7 is detected in dilated and atrophic renal tubules [31].

Kidney tissues from patients with IgA nephropathy (IgAN) show positive MMP-7 staining in renal tubular epithelia, fluids in the tubular lumen, and glomerular podocytes [12,21]. The RNA sequencing data from IgAN patients also show a significant increase in the mRNA expression of MMP-7 in the kidneys [32]. MMP-7 protein is increased in both tubular epithelial cells and the tubulointerstitial compartment of human diabetic kidneys [12,33]. Similarly, renal biopsy specimen analysis reveals MMP-7 induction in lupus nephritis and chronic allograft nephropathy [27,29]. Induction of MMP-7 expression has also been observed in the kidneys of patients with FSGS [12,21]. 

### 3.3. Mechanism of MMP-7 Regulation In Vivo

As to the cues responsible for MMP-7 induction in vivo, many studies have pointed to Wnt/β-catenin signaling, which is activated in virtually every kind of nephropathy [21,34,35]. When Wnt ligands bind to their receptors, β-catenin is stabilized and subsequently translocates into the nucleus for binding to the TCF/lymphoid enhancer-binding factor to regulate the transcription of its target genes, including MMP-7 [34,36,37]. Bioinformatics analysis reveals the presence of putative TCF-binding sites in the promoter region of the *MMP-7* gene [21]. Chromatin immunoprecipitation confirms that β-catenin activation promotes the binding of TCF to the *MMP-7* gene promoter, resulting in the expression of MMP-7 in kidney tubular epithelial cells [21]. 

There is also a correlation between MMP-7 and TGF-β in vivo, suggesting that TGF-β may play a role in mediating MMP-7 expression. Both TGF-β1 and MMP-7 are upregulated in streptozotocin-induced diabetic nephropathy rats. Sirtuin 1 (Sirt1) deacetylates Smad4 and inhibits the expression of MMP-7, indicating that the over-activation of TGF-β is related to the excessive acetylation of Smad4, which, in turn, causes MMP-7 induction [38,39]. Along these lines, resveratrol increases the expression of Sirt1, which inhibits MMP-7 and ultimately alleviates renal injury and fibrosis. Furthermore, TGF-β may indirectly augment MMP-7 by activating canonical Wnt signaling [19]. TGF-β also inhibits Dickkopf-1, an antagonist of Wnt, and potentiates the activity of β-catenin, thereby activating Wnt/β-catenin signaling [40,41]. Of interest, a study shows that MMP-7 can further induce TGF-β production via an MMP-7/Syndecan-1/TGF-β autocrine loop [42,43]. 

## 4. MMP-7 As a Biomarker for Kidney Diseases

Early identification and diagnosis are of importance in slowing the progression of kidney disease and preventing its complications [44]. Serum creatinine and blood urea nitrogen (BUN), two widely used markers for the diagnosis of kidney failure, increase only in the advanced stage of nephropathy. Consequently, kidney diseases are usually diagnosed at a later stage, and the implementation of therapeutic interventions is usually delayed. Therefore, there is an urgent need to develop novel biomarkers for early detection and prognostic assessment of kidney disorders [45]. MMP-7 is upregulated in various kidney diseases, and its protein is predominantly distributed in the apical region of tubular epithelial cells and is detected in the fluids present in the tubular lumen [12,21], suggesting that this protein could be secreted to the urine. Therefore, the level of uMMP-7 can be used as a potential non-invasive biomarker of kidney disease. 

### 4.1. uMMP-7 Predicts the Risk of AKI 

AKI is a relatively common disorder among hospitalized patients, occurring in more than 20% of all hospitalizations in large academic hospitals [46]. AKI causes approximately 2 million deaths each year worldwide. Current diagnostic criteria for AKI require changes in serum creatinine and urine output, which are not sensitive and represent delayed markers. Estimated glomerular filtration rate (eGFR) must decline by approximately 50% before any changes in serum creatinine can be detected [47,48]. Therefore, a sensitive and robust biomarker to predict the risk of AKI and its associated outcomes in patients is urgently needed. Over the past decades, there are tremendous efforts in the nephrology community to discover and validate potential biomarkers for AKI. Several biomarkers, such as kidney injury molecule-1 (Kim-1), neutrophil gelatinase-associated lipocalin (NGAL), and TIMP-2/insulin-like growth factor-binding protein-7 (IGFBP7), have been identified. Although some of them are applied to the clinic, they have various limitations. 

There are two prospective cohort studies showing uMMP-7 as a valuable predictor of severe AKI after cardiac surgery [13,49]. In a prospective, multicenter, two-stage cohort study with 721 patients undergoing cardiac surgery, compared with the lowest quartile, a postoperative uMMP-7 level of 22.6 µg/g creatinine in children represents a >36-fold risk of severe AKI, whereas a postoperative uMMP-7 level of >15.2 µg/g creatinine in adults indicates a 17-fold risk of severe AKI [13]. In terms of predicting prognosis, higher uMMP-7 levels shortly after cardiac surgery are associated with an increased risk of acute dialysis or in-hospital deaths among children and adults, as well as longer duration of intensive care unit and hospital stays [13]. uMMP-7 outperforms other biomarkers, including urinary interleukin-18, NGAL, urinary angiotensinogen, albumin-to-creatinine ratio, and TIMP2/IGFBP7 [50,51,52,53], and the area under the receiver operating characteristic curve (AUC) of uMMP-7 is the largest for predicting severe AKI [13]. Of particular interest, uMMP-7 level peaks at 4 h after surgery, whereas the rise in serum creatinine occurs after 24 h in patients [13]. This is of clinical significance because it allows much earlier identification of the patients who have a high risk of developing AKI than serum creatinine. Therefore, uMMP-7 could be a valuable and robust biomarker for predicting the AKI after cardiac surgery. More studies are needed to replicate the results in larger and more diverse populations and in different settings of AKI. 

### 4.2. uMMP-7 As a Biomarker of CKD Progression

Kidney fibrosis is a common outcome of virtually all CKDs [54,55], which is characterized by excessive accumulation and deposition of ECM, leading to tissue scarring. Measuring the extent of renal fibrosis is essential for determining the prognosis of renal outcomes, monitoring CKD progression, and evaluating the therapeutic efficacy of new treatments [55]. Currently, renal fibrosis is assessed only via percutaneous renal biopsy [55,56]. It is necessary to find non-invasive surrogate biomarkers for evaluating the development and progression of kidney fibrosis. Because activation of Wnt/β-catenin is a common feature of fibrotic CKD [57,58,59,60], one would speculate that the activity of renal Wnt/β-catenin signaling parallels the severity of renal fibrosis. Because the uMMP-7 level reflects the activity of renal Wnt/β-catenin [21], it is then reasonable to measure uMMP-7 to estimate the extent of kidney fibrosis. 

In patients with CKD, uMMP-7 levels are found to positively correlate with renal fibrosis scores and have an inverse association with the renal function [12]. Therefore, uMMP-7 levels may serve as a noninvasive biomarker for kidney fibrosis and a predictor for CKD outcomes, as well as monitor the dynamic of fibrosis progression. Consistent with the findings in the kidney, MMP-7 also affects liver and lung fibrosis after a chronic injury. MMP-7 is upregulated in biliary atresia-associated liver fibrosis, and its expression is considered the best strategy to distinguish between cirrhosis and pre-cirrhosis stages [61,62]. Elevated MMP-7 levels are also detected in the peripheral blood of patients with human idiopathic pulmonary fibrosis (IPF) and may be used as a biomarker for predicting disease progression and death [63,64,65,66]. Serum MMP-7 levels are also increased in IPF patients with severe obstructive sleep apnea [67]. It should be pointed out that uMMP-7 is much more robust than serum MMP-7 in predicting kidney injury, as it is mainly produced from the injured tubular epithelium and expressed apically in CKD. In this context, uMMP-7 is particularly suitable for assessing the severity of fibrosis in the kidney, compared to other organs. 

The feasibility and validity of uMMP-7 as a biomarker to predict CKD progression are recently supported by a prospective cohort study for uMMP-7 to serve as a predictor for IgAN progression [15]. The course of IgAN is highly variable and heterogeneous. The clinical manifestations range from benign asymptomatic microscopic hematuria to severe hypertension and progressive CKD, and renal pathological appearances range from normal to different degrees of mesangial cell proliferation [68]. At present, the final diagnosis of IgAN mainly relies on renal biopsy [68]. On the basis of a histological assessment, variables including mesangial hypercellularity (M), endocapillary hypercellularity (E), segmental glomerulosclerosis (S) and tubular atrophy and interstitial fibrosis (T) give rise to MEST score and provide valuable information to prognostication of IgAN [69]. The lack of sensitive and specific surrogate indicators for long-term outcomes makes it challenging to improve treatment options [70]. A recent prospective observational cohort study shows that the level of uMMP-7 may serve as an independent and powerful predictor for IgAN progression, even for those patients who are still in the early stages of IgAN, as defined by an eGFR of ≥ 60 mL/min/1.73 m^2^ [15]. High levels (> 3.9 μg/g of creatinine) of uMMP-7 increase the risk of IgAN progression by 2.7 times in an adjusted analysis [15]. Several earlier studies have suggested numerous possible biomarkers in serum or urine for predicting IgAN progressions, such as galactose-deficient IgA1, auto-antibodies against Gd-IgA1, fibroblast growth factor 23, angiotensinogen, epidermal growth factor, and Kim-1 [71,72,73,74,75,76,77]. Measurement of uMMP-7 level outperforms each of these biomarkers in risk prediction and improves the risk predictive power of a MEST score [15]. Some retrospective follow-up studies also show that an increase in serum MMP-7 levels is associated with a high risk of poor renal outcome and renal fibrosis [74]. In addition, uMMP-7 is also identified to serve as an independent predictor of tissue remodeling and renal interstitial fibrosis in children with CKD [78]. Another study shows that among people with type 2 diabetes and proteinuric DKD, uMMP-7 concentration is strongly associated with disease progression and subsequent mortality [14]. Taken together, these findings indicate that uMMP-7 may hold promise as a noninvasive biomarker for kidney fibrosis and CKD progression. 

## 5. Roles of MMP-7 in Kidney Diseases

Thanks to the availability of MMP-7 knockout mice, we now have a better understanding of the role of MMP-7 in the pathogenesis of kidney diseases. Although the specific action of MMP-7 in different nephropathy models has not been fully clarified, numerous studies have discovered discrete roles of MMP-7 in renal pathophysiology in different settings. Furthermore, the role of MMP-7 may also evolve with different disease types or the time course of kidney disorders. For instance, MMP-7 may be reparative in the early stages of AKI, whereas it may be detrimental as the disease progresses. Table 2 summarizes the roles of MMP-7 in various kidney diseases.

### 5.1. MMP-7 Protects Against AKI

MMP-7 is induced specifically in renal tubular epithelium after AKI, a condition characterized by the death of tubular cells and the infiltration of inflammatory cells to the kidney [80,81,82]. In AKI patients after cardiac surgery, uMMP-7 levels increase rapidly and peak at 4 h, suggesting MMP-7 induction is an early event [13]. Using MMP-7^-/-^ null mice, a recent study shows that MMP-7 has a renal protective effect against AKI as it alleviates injury by reducing the number of dead tubular cells, promoting the proliferation of tubular cells, and suppressing inflammation [11]. Compared with wild-type controls, MMP-7^-/-^ mice display higher mortality, elevated serum creatinine, and more severe histologic lesions after IRI or cisplatin. These changes are accompanied by more prominent tubular cell death and interstitial inflammation in MMP-7^-/-^ kidneys. In a rescue experiment, injection of exogenous MMP-7 protects against kidney injury in MMP-7^-/-^ mice after IRI, confirming a renal protective action of MMP-7. Of note, MMP-7 may only play a protective role in the early stages of AKI because the long-term activation of MMP-7 leads to kidney fibrosis [12].

### 5.2. MMP-7 Promotes Kidney Fibrosis and CKD Progression

CKD is characterized by excessive ECM accumulation and interstitial fibroblast activation [54,83,84,85]. Given its proteolytic potential to degrade ECM proteins, MMP-7 was originally thought to be anti-fibrotic. Surprisingly, it is found that MMP-7 plays a critical role in the development of fibrotic CKD [12]. In mice, the genetic ablation of MMP-7 reduces the fibrotic lesions and ECM accumulation induced by UUO. Knockout of *MMP-7* also preserves E-cadherin protein and inhibits the de novo expression of vimentin in renal tubules of obstructed kidneys, suggesting that MMP-7 may promote EMT in vivo. Although the role of EMT in renal fibrosis remains controversial, partial EMT is an indispensable part of renal fibrosis [57,86]. Along these lines, it appears that MMP-7 plays an important role in the onset and progression of renal fibrosis by impairing the integrity of renal tubular epithelium and causing partial EMT [12]. Loss of E-cadherin mediated by MMP-7 leads to β-catenin liberation and nuclear translocation, which facilitates CKD progression [57,87]. Of note, MMP-7-mediated β-catenin liberation further induces MMP-7 expression, creating a vicious cycle [12]. Furthermore, TGF-β is known to induce MMP-7 expression, consistent with their role in promoting EMT and kidney fibrosis [38,39]. More studies are needed to confirm the detrimental role of MMP-7 in other models of CKD. 

### 5.3. MMP-7 Induces Podocyte Dysfunction and Proteinuria

In glomerular diseases, MMP-7 is specifically induced in glomerular podocytes of the diseased kidney, implying its potential role in regulating podocyte injury and proteinuria. Indeed, a recent study shows that the infusion of MMP-7 protein or injection of *MMP-7* expression vector induces transient proteinuria in normal mice [7], suggesting that MMP-7 can trigger podocyte injury and impair the glomerular filtration barrier. Furthermore, MMP-7^-/-^ mice are protected against proteinuria and glomerular injury induced by either angiotensin II or adriamycin [7]. Consistent with this finding, incubation of isolated glomeruli with MMP-7 ex vivo increases glomerular permeability and causes foot process effacement. These observations illustrate that MMP-7 impairs glomerular filtration and causes proteinuria in vivo. 

CKD progression is characterized by increasingly widespread lesions in different compartments of the kidney parenchyma. There is ample evidence to suggest that the progression of the primary glomerular disease can trigger tubular injury and interstitial fibrosis, but whether tubular injury affects the function of the glomerulus remains ambiguous [88]. Recently, using conditional knockout mice with tubule-specific ablation of β-catenin, we discovered that tubule-derived MMP-7 is a pathological mediator of glomerular damage [7]. MMP-7 is secreted as a soluble protein from the tubules to the glomeruli and mediates the impairment of slit diaphragm integrity, leading to podocyte dysfunction and increased proteinuria [7]. This suggests that MMP-7 is the key mediator of tubular-to-glomerular crosstalk that promotes proteinuria and CKD progression. 

## 6. Mechanisms and Novel Targets of MMP-7 in Kidney Diseases

**Figure 2:**
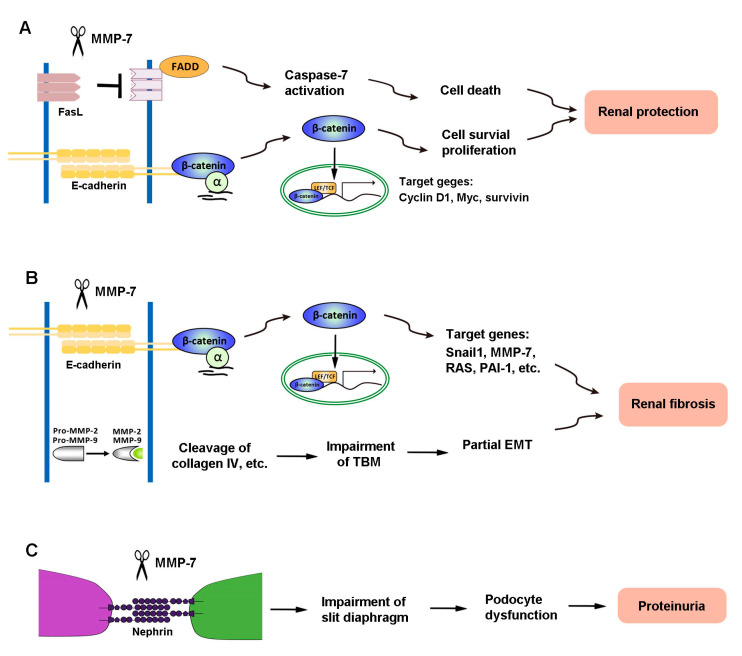
The mechanisms of MMP-7 action in kidney disease. (**A**) In renal tubular epithelial cells, MMP-7 promotes cell proliferation and reduces cell death by degrading E-cadherin and FasL, respectively, and finally plays a role in protecting the kidney during acute kidney injury (AKI). (**B**) However, MMP-7 also degrades E-cadherin and activates proMMP-2 and -9, leading to renal fibrosis. (**C**) In podocytes, MMP-7-mediated degradation of nephrin impairs the integrity of the slit diaphragm, which subsequently causes an increase in proteinuria and eventually leads to renal fibrosis.

The diverse actions of MMP-7 in kidney diseases are presumably mediated by its ability to cleave different substrates. As the primary role of activated MMP-7 is to break down ECM components, MMP-7 is well known to degrade macromolecules, including casein, type I, II, IV, and V gelatins, fibronectin, and proteoglycan [2,89]. Recent findings reveal, however, that MMP-7 is also capable of degrading a multitude of non-ECM substrates, such as FasL, E-cadherin, nephrin, and proMMP-2 and -9 [8,9,10,12]. These novel actions of MMP-7 play a crucial role in mediating its diverse functions in the pathogenesis of kidney disorders. Figure 2 illustrates the mechanisms of MMP-7 action in kidney disease. 

### 6.1. FasL

There are two signaling pathways leading to apoptosis: the intrinsic pathway and extrinsic pathway [90,91,92]. The FasL plays a central role in the death receptor-dependent extrinsic apoptosis pathway [93]. Dependent upon different pathological conditions and different cells, MMP-7 shows a bi-directional effect on the induction or degradation of FasL, which plays distinct roles in regulating cell survival/death in different settings. MMP-7 degrades FasL to decrease the apoptosis of renal tubular cells through FasL/Fas-associated death domain (FADD)/caspase-7 activation, which is one of the mechanisms underlying MMP-7 protection of kidney tubular cells against death in the early stage of AKI [11] (Figure 2). Consistently, MMP-7-induced cleavage of the membrane-bound FasL also plays a role in the process of pulmonary fibrosis [94]. 

Studies also show that MMP-7 affects the fate of renal interstitial fibroblasts in the opposite way. MMP-7 is shown to induce the expression of FasL in cultured fibroblasts, which promotes fibroblast apoptosis and facilitates its resolution after kidney repair following AKI [95]. This observation is supported by studies conducted on cancer cells, which also shows that MMP-7 induces the expression of FasL and subsequently activates the extrinsic apoptotic pathway [10,96]. Furthermore, MMP-7 enhances the staurosporine-mediated intrinsic apoptosis pathway [97,98], leading to fibroblast apoptosis [95]. In this regard, MMP-7-induced fibroblast apoptosis requires the synergistic action of both the intrinsic and extrinsic pathways. At this stage, the mechanism by which MMP-7 induces FasL expression remains to be determined. It is likely that MMP-7 cleaves and activates an unidentified intermediate molecule, which, in turn, induces FasL expression. 

### 6.2. E-Cadherin

E-cadherin, a classical member of the cadherin superfamily, is an epithelial marker that plays an important role in maintaining the integrity of tubular epithelial cells and cell–cell adhesion [99]. It is a calcium-dependent cell–cell adhesion receptor composed of five extracellular cadherin repeats, a transmembrane region, and a highly conserved cytoplasmic tail. MMP-7 is capable of degrading E-cadherin via ectodomain shedding. It has been shown that MMP-7 affects both CKD and AKI by cleaving E-cadherin [8,100]. Loss of E-cadherin impairs the integrity of tubular epithelial cells, which is recognized as the initial step of partial EMT, a process that is essential for tubular atrophy and kidney fibrosis [101,102]. Because E-cadherin and β-catenin associate with each other in a cellular adhesion complex [99,103], the cleavage of E-cadherin would result in the dissociation of the E-cadherin/β-catenin complex, leading to β-catenin liberation (Figure 2). In essence, MMP-7 can induce kidney fibrosis by activating β-catenin in the absence of Wnt ligands [12]. Notably, such β-catenin release caused by MMP-7-mediated degradation of E-cadherin is also found in mouse prostate cancer cells [104]. 

The liberation of β-catenin caused by MMP-7-mediated degradation of E-cadherin provides a rational explanation for why MMP-7 promotes renal fibrosis. The exact same pathway, however, is beneficial and plays a protective role against AKI (Figure 2). E-cadherin maintains tight cell–cell adhesion among tubular epithelia [105]. The ability of MMP-7 to degrade E-cadherin disrupts epithelial cell contact inhibition [106] and leads to cell proliferation [107,108,109]. E-cadherin degradation also leads to β-catenin liberation and downstream signaling. In vitro experiments have confirmed that proliferation-related proteins, such as proliferating cell nuclear antigen (PCNA) and c-Fos, are increased in isolated renal tubules cultured with mitogen-rich serum and MMP-7, compared with serum alone [11]. In short, by cleaving E-cadherin and releasing β-catenin, MMP-7 creates an environment conducive to cell proliferation and protects renal tubules against AKI [11]. However, loss of cell contact inhibition can also trigger EMT and lead to a profibrotic phenotype after the acute injury has resolved. Therefore, MMP-7 induction could be beneficial in the early phase of kidney injury but not in the late stage of chronic kidney disorders. The balance of beneficial versus detrimental effects may rely upon the time point at which MMP-7 activation occurs. 

### 6.3. Nephrin

Nephrin is a key component of the slit diaphragm, which connects adjacent podocyte foot processes and plays a fundamental role in glomerular filtration [110]. We recently demonstrated that nephrin is a specific and direct substrate of MMP-7 [7] (Figure 2). MMP-7 is shown to degrade nephrin in cultured glomeruli, cultured cells, and cell-free systems, which is dependent on its proteolytic activity. Such action of MMP-7 on nephrin degradation is rapid, starting as early as 5 min after incubation. Furthermore, the action of MMP-7 on nephrin appears direct, cleaving purified recombinant nephrin protein in a cell-free system. It should be stressed that the action of MMP-7 is specific, as other MMPs, such as MMP-2 and MMP-9, are unable to degrade nephrin in the same conditions. These findings not only identify nephrin as a novel substrate of MMP-7 but also offer a mechanistic insight into the role of MMP-7 in the development of proteinuria and glomerular lesions [7]. Therefore, MMP-7-mediated cleavage of nephrin impairs slit diaphragm integrity to promote podocyte injury and proteinuria. 

### 6.4. Pro-MMP-2 and -9

MMP-7 also promotes renal fibrosis by proteolytically activating MMP-2 and MMP-9 from their latent zymogen forms. In an in vitro experiment, it has been shown that MMP-7 activates pro-MMP-2 by propeptide removal [111]. Earlier studies in tumor cells also show that MMP-7 is a potential activator of pro-MMP-2 [112], through competitively binding to the TIMP-2 from MMP-2/TIMP-2 complexes, leading to the release of functional MMP-2 [113,114]. Because MMP-2 can convert pro-MMP-9 into MMP-9, it is conceivable that MMP-7 can activate MMP-9 by generating active MMP-2 in both direct and indirect ways [113,114,115]. At present, some studies show that MMP-2 and MMP-9 play an important role in the onset and progression of CKD via degrading type IV collagen, promoting partial EMT and mediating a complex interaction with various cytokines [116,117]. Therefore, MMP-7 could promote kidney fibrosis and CKD progression by activating MMP-2 and MMP-9. 

## 7. Conclusion and Perspectives

Over the last several years, our understanding of MMP-7 in the pathogenesis of kidney disease has dramatically improved. MMP-7 is upregulated in AKI, CKD, and glomerular diseases and is predominantly localized in renal tubular epithelia. Recent findings on novel substrates of MMP-7, such as nephrin, have shed new light on its role and mechanisms of action in a wide variety of kidney diseases. By cleaving E-cadherin and FasL, MMP-7 protects the kidney from AKI by priming renal tubular cells for survival and proliferation. The same MMP-7-mediated degradation of E-cadherin in CKD setting, however, promotes tubular EMT and activates β-catenin in a Wnt-independent fashion, leading to kidney fibrosis. MMP-7 can directly degrade nephrin, resulting in podocyte dysfunction and proteinuria. Several clinical cohort studies suggest that uMMP-7 levels may be used as a noninvasive biomarker for predicting AKI prognosis and monitoring CKD progression in patients. 

Although the present studies have provided invaluable insights into the biological functions of MMP-7 in kidney diseases, how to translate this knowledge into patient care is a daunting task. The revelation of MMP-7 upregulation provides hopes for the application of uMMP-7 as a noninvasive biomarker, and future clinical studies for validation in large and diverse populations of patients are warranted. Developing a therapy to target MMP-7 will rely upon highly selective inhibitors for MMP-7, which are not yet available. Future studies may focus on developing efficient strategies to inhibit MMP-7 expression in vivo for modulating the course of kidney disease. We hope that continuing this line of investigation will improve our understanding of the role of MMP-7 and its mechanism of action in the pathogenesis of various kidney disorders, and eventually translate into improved patient care. 

## Figures and Tables

**Table 2:** Roles of MMP-7 in kidney diseases.

Disease	Role of MMP-7	Ref.
AKI ^1^	Protecting against AKI by priming tubular cells for proliferation and survival	[11]
UUO ^2^	Promoting renal fibrosis by activating partial EMT and β-catenin	[12]
Proteinuric CKD ^3^	Increasing urinary albumin excretion by impairing the glomerular filtration barrier	[7]
Diabetic nephropathy	Initiating diabetic nephropathy by expanding glomerular mesangium and thickening glomerular basement membrane	[79]
Chronic allograft nephropathy	MMPs, including MMP-7, contribute to the deregulation of extracellular matrix remodeling and possibly EMT.	[29]
Light chain deposition disease	The decrease of MMPs, including MMP-7, leads to the accumulation of tenascin and extracellular matrix	[30]
Amyloid light- chain amyloidosis	The increase of MMPs, including MMP-7, leads to the reduction of extracellular matrix	[30]
Renal cell carcinoma	Affecting tumor progression by regulating invasion and angiogenesis	[22]

^1^ Acute kidney injury. ^2^ Unilateral ureteral obstruction. ^3^ Chronic kidney disease.
